# Summary measures of socioeconomic and area-based inequalities in fertility rates among adolescents: evidence from Ethiopian demographic and health surveys 2000–2016

**DOI:** 10.1186/s12889-021-10729-5

**Published:** 2021-04-21

**Authors:** Sanni Yaya, Edward Kwabena Ameyaw, Dina Idriss-Wheeler, Gebretsadik Shibre, Betregiorgis Zegeye

**Affiliations:** 1 School of International Development and Global Studies, Faculty of Social Sciences, University of Ottawa, Ottawa, Canada; 2 The George Institute for Global Health, Imperial College London, London, UK; 3 School of Public Health, Faculty of Health, University of Technology Sydney, Sydney, NSW Australia; 4 Interdisciplinary School of Health Sciences, Faculty of Health Sciences, University of Ottawa, Ottawa, Canada; 5 Department of Reproductive Health and Health Services Management, School of Public Health, Addis Ababa University, Addis Ababa, Ethiopia; 6 HaSET Maternal and Child Health Research Program, Shewarobit Field Office, Shewarobit, Ethiopia

**Keywords:** Adolescent fertility rate, Inequality, Reproductive health, Global health, Ethiopia, DHS

## Abstract

**Background:**

One of the highest rates of adolescent pregnancies in the world is in sub-Saharan Africa. Most adolescent pregnancies in the region are unintended or unwanted, due to poor access to information and services on sexual and reproductive health for adolescents. Ethiopia has high adolescent fertility rates (AFR) with disparities across socioeconomic subgroups and regions. This study assessed the magnitude and trends of socioeconomic and area-based AFR inequalities in Ethiopia.

**Methods:**

The 2000 and 2016 Ethiopia Demographic and Health surveys (EDHS) was analyzed using the World Health Organization’s (WHO) Health Equity Assessment Toolkit (HEAT) software. Adolescent fertility rates were disaggregated using three equity stratifiers (economic status, education and residence) and analyzed through four summary measures (Difference (D), Population Attributable Risk (PAR), Ratio (R) and Population Attributable Fraction (PAF)) to assess inequality. To measure statistical significance, point estimates were constructed using a 95% Uncertainty Interval (UI).

**Results:**

Large socio-economic and urban-rural inequalities were observed within the 16-year period. Adolescents in less well-off socio-economic groups (PAF: -62.9 [95% UI; − 64.3, − 61.4], D: 96.4 [95% UI; 47.7, 145.1]), uneducated (R: 8.5 [95% UI; 4.8, 12.2], PAR: -76.4 [95% UI;-77.7, − 75.0]) and those from rural areas (D: 81.2 [95% UI; 67.9, 94.6], PAF: -74.2 [95% UI, − 75.7, − 72.7]) had a higher chance of pregnancy and more births than their counterparts.

**Conclusions:**

Socioeconomic (education and economic status) and place of residence determine adolescents’ pregnancy and childbearing. Policies and programs should be directed at preventing child marriage and early fertility so that adolescents continue to access education, sexual and reproductive health care as well as access employment opportunities. More emphasis should be placed on subpopulations with disproportionately higher adolescent pregnancy and childbirth.

## Background

Sub-Saharan Africa has one of the largest populations of adolescent girls in the world, many of whom are exposed to unintended pregnancy [1, 2]. Adolescent Fertility Rate (AFR) is defined as the “number of births per 1,000 women aged 15 to 19” [3, 4] and remains high in the World Health Organisation (WHO) African Region; 99.1 per 1000 compared with a global average of 43.9 per 1000, over the period 2007–2016 [5]. Inadequate sex education along with insufficient access to contraception and reproductive health services are generally implicated in most adolescent pregnancies [6, 7], but new findings show that several other complex socioeconomic and cultural factors can also contribute to increased likelihood of adolescent pregnancy [8, 9].

Close to 11% of births worldwide are children born to nearly 16 million adolescents between the ages of 15 to 19 [6]. Despite the global reduction in adolescent fertility in recent decades [7, 10, 11], it is estimated that 21 million girls 15 to 19 years old and 2 million girls under 15 years become pregnant every year in low and middle-income countries (LMICs) [6]. Unfortunately, births to adolescents and the associated negative outcomes continue to be serious problems in many LMICs [6].

Pregnancy among adolescent females is a major public health concern because of complicated health outcomes for young mothers and newborns. Globally, the major causes of adolescent girls’ death are pregnancy and childbirth related complications [12]. LMICs account for 99% of maternal death for females aged 15–19 years [12]. Each year, almost 4 million adolescents are exposed to unsafe abortions, contributing to maternal morbidity and mortality [13].

Furthermore, pregnancy induced hypertension, puerperal endometritis and sepsis risk are higher among adolescent females aged 10–19 years in comparison to women who are between 20 and 24 years of age [14]. Other complications associated with adolescent pregnancy include premature delivery, stillbirth, fetal distress, birth asphyxia, low birthweight, and miscarriage [14]. Many of the girls with unwanted pregnancies are unmarried and have to deal with the pervading stigma of adolescent pregnancies and do not seek necessary healthcare [15]. There is an increased likelihood of dropping out of school for adolescents who are pregnant, which further exacerbates women’s existing economic disadvantage [16].

Determinants of adolescent pregnancy identified in previous studies include low socioeconomic status (i.e., low-level of education, low income) as well as inadequate sex education and access to contraception and reproductive health services [17]. Furthermore, adolescent pregnancy is higher in rural regions where cultural norms and practices have influence [17–21].

Ethiopia is a sub-Saharan African country with an estimated 6 million adolescent girls [22] and the second largest adolescent population in Africa. The global adolescent fertility rate of 43.9 per 1000 [23] is about a third of the fertility rate of Ethiopian adolescents aged 15–19 years which is estimated at 130 births per 1000 [24]. The government of Ethiopia flagged adolescent pregnancy and childbirth as an important public health concern through its health sector transformation plan [25] and national reproductive health strategy [26]. More specifically, the initiative proposes to strengthen adolescent, and youth focused reproductive health services; the intention is to reduce the high rates of abortions, fistulas and other pregnancy-related complications [25].

The 2016 Ethiopian Demographic and Health Survey (EDHS) reported that 13% of adolescent girls are pregnant or became a mother for the first time [24]. However, disparities remain as certain groups are particularly vulnerable and at risk for pregnancies. For instance, the adolescent fertility rate among rural adolescent girls was 15%, whereas among urban adolescent girls, it was 5%. Similarly, 28% of pregnant adolescents had no education compared to 3% with secondary education. Pregnancy rates were also higher for rural (e.g., 23% in Affar) compared to urban adolescents (e.g., 3% in Addis Ababa) [24]. Despite these identified inequities, there are few studies on determinants of adolescent pregnancy and childbirth in Ethiopia [27, 28].

While high-quality evidence on the magnitude of disparities in AFR is critically needed to plan equity sensitive interventions, to our knowledge, no study has yet investigated this issue in the context of Ethiopia. This study examined socioeconomic and urban-rural inequalities in AFR in Ethiopia and considered the trends of inequality over the 16-year period between 2000 to 2016.

## Methods

### Data source

Cross-sectional data from the EDHS for the periods of 2000, 2005, 2011 and 2016 were used for this study. The Central Statistical Agency (CSA) of Ethiopia conducted the nationally representative household surveys which house data on various health indicators including adolescent fertility rates in the country. The EDHS takes place every 5 years with four rounds since its inception in 2000. There have been two interim EDHSs conducted in 2014 and 2019; the latter had not been released at the time of this study and is not included in the analysis.

### Study population and sample

The survey covered adolescent girls aged 15 to 19 years. Eligible adolescents included those who were permanent residents of included households or those who joined the households the night before the surveys. Ethiopia is estimated to have about 6 million adolescents [22]. A total of 64,506 adolescents were included in this study from the 2000 (*n* = 16,182), 2005 (*n* = 15,406), 2011 (*n* = 17,461) and 2016 (*n* = 15,457) EDHSs.

### Measures of inequality

Inequality is a multifaceted concept but could be explained as the state of not being equal, especially in status, rights, and opportunities [29]. Adolescent fertility rate (AFR) - the inequality variable in this study **-** is the proportion of births per 1000 adolescents between the ages of 15–19 years. The AFR was disaggregated and reported as a percentage across socioeconomic and urban-rural subpopulations by three dimensions of inequality - economic status, education and place of residence – for the four EDHS years studied. Economic status is approximated using a wealth index. In the DHS, wealth index is a composite variable that employs Principal Component Analysis by using different characteristics such as asset ownership [e.g. bicycle, television], sanitation facilities and materials used for house construction to classify participants into five groups: poorest, poor, middle, rich and richest [30]. Participant educational status was categorized as no-education, primary, secondary or more, while place of residence was either urban or rural. Educational status and wealth are ordered equity stratifiers (i.e., have a natural ordering), while place of residence is a non-ordered equity stratifier. The choice of summary measures (described below) depends on the type of equity stratifier. For instance, the summary measure for educational status (ordered/ranked equity stratifier) and for place of residence or sex (non-ordered equity stratifier) may differ [31].

### Statistical analysis

Using the 2019 updated version of the WHO’s Health Equity Assessment Toolkit (HEAT) software [31], we analyzed the socioeconomic and urban-rural inequalities in AFR in Ethiopia over 16 years (2000 to 20,016). Raj and Boehmer [13] provide a complete description of the WHO HEAT software and its access [15].

AFR was disaggregated using a combination of absolute (Difference [D] and Population Attributable Risk [PAR]) and relative (Ratio [R] and Population Attributable Fraction [PAF]) summary measures calculated for each of the three equity stratifiers (economic status, education and place of residence). Hosseinpoor et al., 2016 provide a detailed description of the summary measures’ calculations (i.e. formulas) and properties (i.e. definitions) as well as how to interpretate the results [31].

Simple measures (D and R) were used to show absolute difference and relative ratio for a dimension with (i) two categories (place of residence), and (ii) multiple categories with an identified reference subgroup (wealth index and education). For complex measures of PAF and PAR, positive and negative values signal favorable or adverse health indicators, respectively. AFR is considered an adverse indicator and rendered negative values. Furthermore, a value of zero for PAF and PAR indicated absence of inequality while a greater absolute value signaled a higher level of inequality. For simple measures used in this study, a value of zero [0] for difference and a value of one [1] for ratio indicated absence of inequality. To measure statistical significance of measures between the subgroups as well as determine if inequality changed over the 16-year period (2000 to 2016), point estimates were constructed using a 95% Uncertainty Interval (UI) for each measure for each year of the surveys. To conclude that a statistically significant AFR disparity exists in the sub-groups of each equity stratifier, the lower and upper bounds of the 95% UIs must not include 0 for D, PAR and PAF, or 1 for R. To determine existence of inequality, change over time, the UIs must not overlap. Complete descriptions of R, D, PAF and PAR calculations as they pertain to AFR can be found in Yaya et al., 2020 [32]. Strengthening Reporting of Observational studies in Epidemiology (STROBE) reporting guidelines was used to organize the findings [19].

### Ethical consideration

The DHS Program follows ethical standards for ensuring the protection of respondents’ privacy and EDHS protocols were reviewed and approved by Inner City Fund (ICF) International Institutional Review Board (IRB) as well as an IRB in the host country. Informed consent and survey implementation were completed by The Central Statistical Agency in Ethiopia with technical support and assistance from ICF. Participants were guaranteed confidentiality and anonymity (i.e., a series of de-identification steps to ensure participant responses are anonymized are described in detail here: http://goo.gl/ny8T6X). WHO HEAT was developed using free and open-source software R and the analytical software is pre-installed with WHO Monitor database that draws from DHS data and Multiple Indicator Cluster Survey (MICS) data from 94 countries [31]. It is an open access, free, publicly available online (or downloadable offline) analytical tool for researchers without necessary permissions because the secondary raw data is also publicly available through the DHS Program and pre-installed (https://www-who-int.proxy.bib.uottawa.ca/data/gho/health-equity/assessment_toolkit).

## Results

### Line graph using disaggregate data

**Fig. 1:**
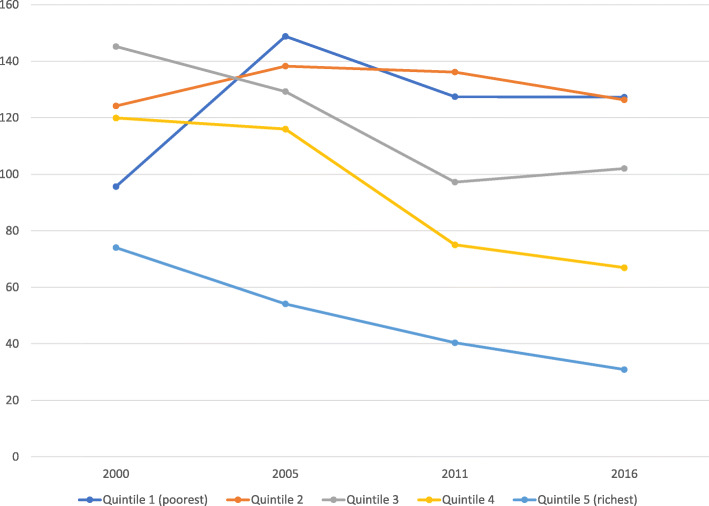
Trends in AFR by wealth quintiles: Evidence from Ethiopia demographic and health survey (2000–2016)

The graphical analysis showed that AFR among the richest women declined over the 16-year period but was highest among women in the penultimate wealth quintile in 2005 (see Fig. 1).

### Trends in AFR by educational status

**Fig. 2:**
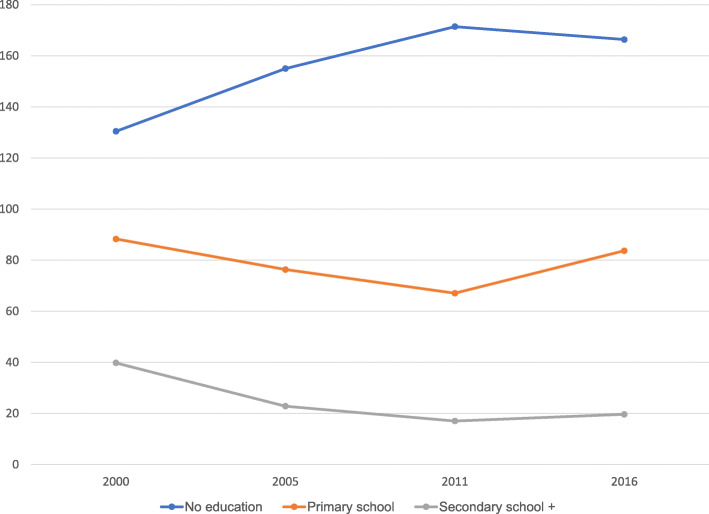
Trends in AFR by educational status: Evidence from Ethiopia demographic and health survey (2000–2016)

Analysis of AFR by educational status indicated that throughout the period studied, women who had no formal education have higher AFR whilst those with secondary or higher education had the least rate (see Fig. 2).

### Trends in AFR by place of residence

**Fig. 3:**
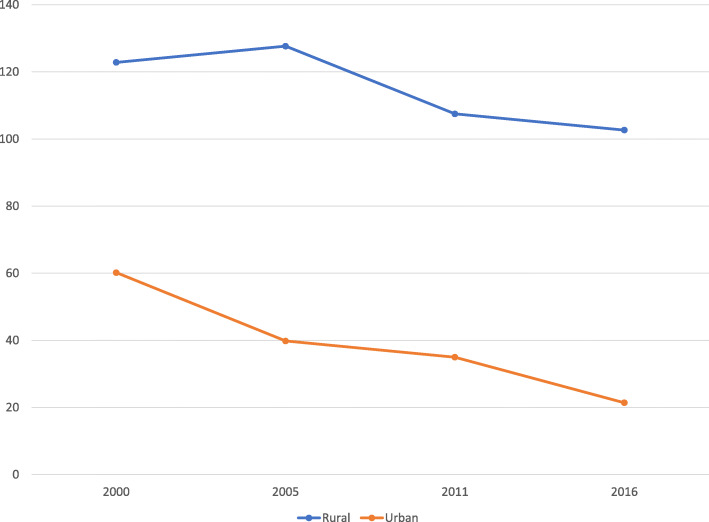
Trends in AFR by place of residence: Evidence from Ethiopia demographic and health survey (2000–2016)

We realized AFR dominated among rural residents between 2000 and 2016 (Fig. 3).

### Adolescent fertility rate across socioeconomic and area-based subgroups

The national AFR was 109.6, 109.1, 87 and 83.1 percentage points in 2000, 2005, 2011 and 2016, respectively. The current study showed disparities in AFR across socioeconomic and place of residence over the 16 years in Ethiopia. For instance, in 2005, women in the richest quintile had significantly lower AFR as compared to the other quintiles, while no difference was observed among the other quintiles. AFR was variable across educational status-subgroups in all four surveys, with a significantly higher fertility rate among adolescents with no education subgroups followed by primary school subgroups as compared to secondary school and above, respectively. There were differences in AFR among rural and urban residents in all four surveys with significantly higher rates among rural residents (Table 1).

**Table 1:** Magnitude and trends of Adolescent Fertility Rate (AFR) across socioeconomic and area-based subpopulations in Ethiopia from 2000 to 2016

Dimension of inequalities	Year							
	2000		2005		2011		2016	
	Number of births per 1000 women aged 15–19 years) (95% UI)	Popn	Number of births per 1000 women aged 15–19 years) (95% UI)	Popn	Number of births per 1000 women aged 15–19 years) (95% UI)	Popn	Number of births per 1000 women aged 15–19 years) (95% UI)	Popn
**Economic status**								
Quintile 1 (poorest)	95.6 (77.4, 117.6)	2392	148.8 (114.5, 191.1)	2233	127.5 (96.9, 165.9)	2780	127.3 (86.7, 183.0)	2435
Quintile 2	124.2 (99.0, 154.6)	2956	138.3 (110.5, 171.7)	2772	136.1 (103.3, 177.4)	3020	126.3 (89.4, 175.6)	2778
Quintile 3	145.2 (120.0, 174.7)	3245	129.3 (96.2, 171.6)	3152	97.2 (73.8, 127.0)	2907	102.0 (75.5, 136.5)	2774
Quintile 4	119.9 (92.3, 154.4)	3168	116.0 (91.2, 146.4)	2674	75.0 (54.4, 102.7)	3415	66.9 (48.6, 91.5)	3053
Quintile 5 (richest)	74.0 (51.4, 105.6)	4419	54.1 (41.2, 70.7)	4574	40.4 (28.3, 57.2)	5337	30.9 (22.1, 43.0)	4415
**Education**								
No education	130.4 (118.9, 142.9)	10,689	155.0 (129.3, 184.7)	8149	171.4 (137.3, 211.9)	4902	166.4 (123.1, 221.0)	3193
Primary school	88.3 (67.2, 115.2)	3332	76.3 (59.5, 97.4)	4711	67.0 (54.0, 83.0)	9312	83.7 (65.5, 106.3)	8003
Secondary school +	39.8 (25.2, 62.1)	2160	22.8 (16.07, 32.3)	2545	17.0 (10.0, 28.9)	3246	19.6 (14.2, 27.1)	4260
**Residence**								
Rural	122.8 (111.9, 134.7)	12,778	127.7 (115.2, 141.3)	12,150	107.5 (96.3, 119.8)	12,540	102.7 (91.5, 115.0)	11,739
Urban	60.2 (45.1, 79.9)	3404	39.8 (31.3, 50.5)	3256	35.0 (25.7, 47.4)	4921	21.4 (15.9, 28.8)	3718

**Source:** 2000, 2005, 2011, 2016 EDHS; UI: Uncertainty Interval; Popn: Population

### Extent and trends in AFR inequalities across different dimensions

Table 2 shows absolute and relative socioeconomic and area-based inequalities in AFR in Ethiopia from 2000 to 2016. Substantial absolute and relative wealth-driven inequality in AFR were observed in all four survey years by simple (D, R) and complex (PAF, PAR) measures, except in 2000 for the Difference and Ratio measure. The PAR measure in 2000, 2005, 2011 and 2016 was  − 35.6% (95% CI: − 36.6, − 34.6), − 55% (95% CI: − 56.2, − 53.8), − 46.6% (95% CI: − 47.8, − 45.5) and − 52.2% (95% CI: − 53.5, − 51.0), respectively.

**Table 2:** Extent and trends of socio-economic and area-based inequality in Adolescent Fertility Rates (AFR) in Ethiopia from 2000 to 2016

Dimension	Year				
		2000	2005	2011	2016
	Measure	%(95%UI)	%(95%UI)	%(95%UI)	%(95%UI)
Economic status	D	21.6 (−11.7, 54.9)	94.7 (53.9, 135.5)	87.1 (50.0, 124.2)	96.4 (47.7, 145.1)
	PAF	−32.5 (−33.4, −31.6)	−50.4 (−51.5, − 49.3)	−53.6 (−54.9, −52.3)	−62.9 (−64.3, −61.4)
	PAR	−35.6 (−36.6, −34.6)	−55.0 (−56.2, − 53.8)	−46.7 (−47.8, −45.5)	−52.2 (− 53.5, − 51.0)
	R	1.3 (0.75, 1.82)	2.8 (1.7, 3.8)	3.2 (1.8, 4.6)	4.1 (2.1, 6.2)
Education	D	90.7 (69.1, 112.2)	132.2 (103.4, 160.9)	154.4 (116.1, 192.7)	146.7 (97.6, 195.8)
	PAF	−63.7 (−65.5, −62.0)	−79.1 (−80.5, −77.6)	−80.4 (−81.9, −79.0)	−76.4 (−77.7, −75.0)
	PAR	−69.9 (−71.8, −67.9)	−86.3 (−87.9, −84.7)	−70.0 (−71.3, −68.8)	−63.5 (− 64.6, − 62.3)
	R	3.28 (1.8, 4.8)	6.8 (4.1, 9.5)	10.1 (4.3, 15.8)	8.5 (4.8, 12.2)
Place of residence	D	62.7 (42.0, 83.3)	87.9 (71.7, 104.0)	72.5 (56.6, 88.4)	81.2 (67.9, 94.6)
	PAF	−45.1 (−46.4, −43.8)	−63.5 (−64.9, −62.1)	−59.8 (−61.2, −58.5)	−74.2 (−75.7, −72.7)
	PAR	−49.5 (− 50.9, −48.1)	−69.3 (− 70.8, −67.8)	−52.1 (− 53.3, − 50.9)	−61.7 (− 62.9, −60.5)
	R	2.0 (1.4, 2.7)	3.2 (2.4, 4.0)	3.1 (2.1, 4.1)	4.8 (3.3, 6.3)

The pattern of wealth-driven inequality remained constant by simple measures (D, R) and fluctuated by the PAR measure over time. However, the PAR findings indicated an increased trend between the first and the last survey points. The PAF measure showed a steady rise over the 16 years.

Similarly, absolute and relative educational status inequality in AFR was observed in all surveys by both simple (D, R) and complex (PAF, PAR) measures. While Difference showed a constant pattern overtime, ratio showed some fluctuation with an overall increasing trend between the first and last years. The PAF pattern increased from 2000 to 2005 and decreased from 2011 to 2016. Based on the PAR, the disparity widened between 2000 and 2005, and then reversed thereafter until 2016.

Results also showed significant absolute and relative residence related inequality in AFR. The PAR and PAF followed the same trend; a rise between 2000 and 2005, a decline between 2005 and 2011, followed by a rise thereafter, with an overall increase between the first and the last survey periods (Table 2).

## Discussion

This study investigated the magnitude and trend over time of inequality in AFR over a 16-year period (2000–2016) in Ethiopia. Using WHO’s HEAT software, the summary measures and equity stratifiers in this study illustrated the the existence of economic status, education and place of residence inequalities in AFR in all four EDHS years. Findings revealed that adolescent girls in the lower socioeconomic gradient and rural adolescents had a higher chance of AFR than their counterparts. Patterns of disparities in AFR within the 16 years were different for each summary measure and need contextual interpretation.

As seen in other studies [1, 27, 28], there was significant wealth-based inequality in all survey years by all the four measures with higher concentration of AFR reported among the poorest subpopulations. While it is essential for policymakers to ensure wealth is distributed fairly across societies by creating opportunities for everyone, unfortunately, only a small and privileged subgroup benefits, which seems to also be true for the context in Ethiopia [33]. Essentially, vulnerable communities where there is increased poverty, lack of education, little access to modern contraceptives as well as sexual violence tend to experience higher rates of adolescent pregnancy [19]. Adolescents from wealthy households tend to have employment and therefore, they can access modern contraceptives and increase likelihood of delayed childbearing [34]. Similar reports have been found in Latin American and the Caribbean [35] as well as across LMICs [36].

The level of education was associated with inequalities in AFR across all surveys over the 16 year-period, despite some fluctuations. This wide variation over different subpopulations appears to weaken the country’s commitment to equity and inclusion. The PAR findings show that if there were no education related differences in the population studied, then the national AFR would have decreased by 70% in 2000 and 63.5% in 2016. Even though there has been a decrease in AFR between educated and non-educated adolescents over the last several years, the rates are still higher, particularly in certain sub-groups, and therefore interventions must continue to prevent pregnancy among adolescent females and its adverse maternal and neonatal health outcomes.

Infants born to adolescents have higher risks of low birth weight, preterm delivery and severe neonatal conditions [14]. Rapid repeat pregnancy, which is prevalent in some settings, is a challenge for young mothers because of increased health risks for both mother and child [16]. Similar to other work, findings in this study suggest socioeconomic conditions are associated with the disparities observed in AFR [37–39]. Adolescent females with little or no education are not as likely to negotiate safe sex; therefore, increased education may positively contribute to their bargaining power, increased knowledge, better access to resources, and greater autonomy in making decisions [37]. In addition, adolescent sexual debut appears to be associated with level of education, such that education decreases the likelihood of exposure to pregnancy. Findings from a 2018 study in Norway showed that an additional year of education reduced the likelihood of adolescent pregnancy by eight percentage points [38]. Investing in education of female adolescents can provide women with the ability to make informed decisions regarding the number of children she wants to have and the ability to control timing of pregnancy through availability of modern contraceptives and the knowledge of how to use them [39].

Adolescent girls with unintended pregnancies turn to underground, unsafe abortions in the midst of prevailing restrictive abortion laws, as seen in Nigeria, which increase chance of suffering severe complications and death [40]. Plausibly, rural and poor adolescents of Ethiopia share similar experiences due to their relatively disadvantaged status. Adolescent friendly maternity services including family planning services are required to assist these adolescents. There is a lack of access to contraception information and services, safe abortion and skilled pregnancy care even though many adolescent girls are sexually active [41]. This is most likely due to socio-cultural norms in many rural communities where stigma exists surrounding sex, sexuality, abortion, contraception and pregnancy among young people. Furthermore, there is gender inequality related to adverse social perceptions, the brunt of which is born by adolescent girls as compared to boys. If left unchallenged, this equity issue will continue to imperil women’s wellbeing [42].

The existence of residential disparities in AFR was identified in the current study in all four surveys and by all four measures. Other studies from sub-Saharan Africa had similar findings [1, 2, 34, 43]. In the most recent survey (2016 EDHS), the PAR measure indicated higher pro-rural absolute area-based inequality in AFR. Similarly, the ratio measure in the same survey concluded that, AFR in rural regions was 4.7 times higher than urban regions [24].

Community support, supportive social networks and a positive environment are important predictors of adolescents’ health-seeking practices [44, 45]. The value of socio-cultural norms must not be underestimated when looking at influencing sexual and healthcare seeking behaviours; engaging communities through gatekeepers (i.e. parents, elder, community leaders) is central to creating successful interventions [8]. Our findings suggest that engagement with gatekeepers is a key consideration to reduce AFR, particularly in rural locations and among uneducated and/or poor adolescents of Ethiopia.

The study has several strengths. To the best of our knowledge, this work is the first to examine inequalities in AFR in Ethiopia using data from four EDHS. Findings from this study can guide both policy and future research AFR in Ethiopia. Several summary measures were used for each dimension of inequality studied, contributing to increased rigor in the approach and quality of findings. The weaknesses of each summary measure are enhanced by the strengths of the other measures providing four measures for each dimension of inequality. The use of relative and absolute inequality measures allowed investigation of both extent and trends of AFR inequality in Ethiopia from multiple perspectives. The subgroup analysis of AFR inequality identify possible policy and program foci for the Ethiopian government, particularly in relation to the adolescent sexual and reproductive health specific UN Sustainable Development Goals (SDGs). This study used the WHO health equity monitor database which houses established DHS program data, strengthening the quality of conclusions drawn from the study.

Nonetheless, the study has some limitations. It is a descriptive study of AFR inequality using the WHO HEAT Software outlining inequity across specific dimensions but does not allow for an in-depth assessment of the impact of other factors which underlie the observed inequality. The latter would require a decomposition technique. Studies in the future could apply this statistical method to discern how each determining factor impacts AFR disparity. In addition, the cross-sectional nature of DHS data does not allow inference of causation from significant associations. As a secondary database, the authors do not have control over the selection and measurement of the variables in DHS data and subsequent WHO health equity monitor database [46]. Finally, while both relative and absolute measures have been advocated by international bodies such as the WHO for monitoring health inequalities, they cannot substitute for analytical evaluation of inequality such as decomposition techniques.

## Conclusions

This study illustrated the inequality in AFR in Ethiopia across three dimensions – education, economic status and place of residence. Findings indicated that adolescent girls from wealthy households and those living in urban areas were at an advantage while also demonstrating a stable inequality pattern over the period of the study. Policy makers need to formulate strategies for the disadvantaged subgroups (i.e., low socioeconomic status and rural) which carry a disproportionate burden of inter-generational maternal and newborn morbidity and mortality. Proactive strategic planning (i.e., increased education, access to safe modern contraception) will contribute to positive achievements of and opportunities for the adolescents, while also reaching maternal mortality related SDGs. Potential risks of AFR (i.e., psychological problems, chronic illness, adolescent maternal and child morbidity) could be prevented if programs and policies are targeted so they improve conditions of underprivileged adolescents.

## Data Availability

The datasets generated and/or analyzed during the current study are available in the WHO’s HEAT version 3.1 [https://www.who.int/gho/health_equity/assessment_toolkit/en/].

## Abbreviations

AFR: Adolescent Fertility Rate
D: Difference
DHS: Demographic and Health Survey
EA: Enumeration Area
EDHS: Ethiopia Demographic and Health surveys
HEAT: Health Equity Assessment Toolkit
HEMD: Health Equity Monitor Database
PAR: Population Attribuable Risk
R: Ratio
SDG: Sustainable Development Goals
UI: Uncertainty Interval
WHO: World Health Organization
